# A machine learning approach toward automating spatial identification of LAG3+/CD3+ cells in ulcerative colitis

**DOI:** 10.1038/s41598-023-49163-5

**Published:** 2023-12-08

**Authors:** Edward D. Bonnevie, Eric Dobrzynski, Dylan Steiner, Deon Hildebrand, James Monslow, Mohan Singh, Vilma Decman, David L. Krull

**Affiliations:** 1 Cellular Biomarkers, GSK, Upper Providence, USA; 2 NCS-Pathology, GSK, Stevenage, UK

**Keywords:** Biomarkers, Colitis, Fluorescence imaging

## Abstract

Over the past decade, automation of digital image analysis has become commonplace in both research and clinical settings. Spurred by recent advances in artificial intelligence and machine learning (AI/ML), tissue sub-compartments and cellular phenotypes within those compartments can be identified with higher throughput and accuracy than ever before. Recently, immune checkpoints have emerged as potential targets for auto-immune diseases. As such, spatial identification of these proteins along with immune cell markers (e.g., CD3^+^/LAG3^+^ T-cells) is a crucial step in understanding the potential and/or efficacy of such treatments. Here, we describe a semi-automated imaging and analysis pipeline that identifies CD3^+^/LAG3^+^ cells in colorectal tissue sub-compartments. While chromogenic staining has been a clinical mainstay and the resulting brightfield images have been utilized in AI/ML approaches in the past, there are associated drawbacks in phenotyping algorithms that can be overcome by fluorescence imaging. To address these tradeoffs, we developed an analysis pipeline combining the strengths of brightfield and fluorescence images. In this assay, immunofluorescence imaging was conducted to identify phenotypes followed by coverslip removal and hematoxylin and eosin staining of the same section to inform an AI/ML tissue segmentation algorithm. This assay proved to be robust in both tissue segmentation and phenotyping, was compatible with automated workflows, and revealed presence of LAG3^+^ T-cells in ulcerative colitis biopsies with spatial context preserved.

## Introduction

Inflammatory diseases affect a large portion of the population and have a significant impact on the quality of life of patients^1^. One such disease is ulcerative colitis (UC), a condition where T-lymphocytes (T-cells) cause chronic mucosal inflammation^2^. Several treatment options exist that target inflammatory signaling (e.g., the anti-TNF-α drug adalimumab); however, these treatments are not effective in all patients and other treatment options are warranted^3^. Recent evidence suggests that lymphocyte activation gene-3 (LAG3) is associated with inflammatory signaling and the resulting mucosal inflammation in UC^4^. Specifically, the prevalence of CD3^+^/LAG3^+^ T-cells is increased in UC, with the number of CD3^+^/LAG3^+^cells in inflamed UC greater than those observed in non-inflamed UC, which in-turn is increased over healthy controls. LAG3 is a transmembrane protein upregulated on newly activated T-cells (e.g., CD4^+^ and CD8^+^ T cells) with increased co-expression on CD4^+^ cells rather than CD8^+^ cells^5^. In a similar fashion to PD1 and CTLA-4, LAG3 can function as an inhibitory co-receptor that binds to MHC class II^6^. Due to its prevalence in active inflammatory signaling in UC, LAG3 has therefore emerged as a therapeutic target in clinical investigation^7^.

Clinical trial endpoints have been supported by histology for decades. Classically, these endpoints have been informed by pathologist assessment of H&E-stained sections from trial biopsies. More specific to biological targets, immunohistochemical (IHC) stained slides using chromogenic stains have been used to identify specific cell types of interest. More recently, IHC stains have been developed to identify druggable targets, and during clinical trials, these assays can shed light on treatment responders versus non-responders. Additionally, as such assays are further developed and implemented in both research and clinical trials, they can prove useful for patient stratification and selection. Finally, as treatments emerge as a standard of care and the assay can predict patient response, some of these assays can be approved as a companion diagnostic assay. These assays are emerging as indispensable tools in determining the best treatment for a specific patient^8^. In line with these previous efforts, the objective of this study was specifically to identify and quantify LAG3+ T-cells in ulcerative colitis samples with their spatial context preserved.

In addition to IHC staining, immunofluorescence (IF) staining has emerged as a gold standard for histology-based assays in many research labs. While dual (and higher plex) chromogen based IHC enables simultaneous detection of multiple markers on a single tissue section, the use of IF has enabled robust identification of additional markers on single sections, in some cases 7 or more markers^9^. The advantage of IF over IHC is readily evident in digital image analysis. Chromogen-based detection of multiple markers is traditionally captured in a single brightfield image, and subsequent deconvolution analyses are necessary to isolate individual stains^10^. This post-hoc image processing of stain deconvolution can be effective but maintains a degree of complexity when two or more markers may overlap. In IF analyses, image files are constructed as planes of monochromatic images, captured individually through the combination of specific fluorophores with their corresponding spectral wavelength filters. That is, the colors are separated at image acquisition and color deconvolution is not necessary. However, collection of IF images poses alternate limitations compared to brightfield IHC. Visual inspection of a brightfield image allows a trained scientist or pathologist to easily identify tissue sub-compartments due to the tissue texture and morphology that is visible within a brightfield image. In a similar fashion to manual identification or segmentation of tissue compartments, brightfield images can be optimal for computer vision approaches developed for the same task^11^. Further, AI/ML computer vision approaches trained by manual annotation of H&E staining have emerged as a method to enhance throughput and repeatability in image analysis^12^.

Here, we describe development of an analysis pipeline to support ulcerative colitis clinical trials. Specifically, this pipeline includes staining assays and image analysis algorithms that identify the spatial abundance of CD3^+^/LAG3^+^ T-cells. In this pipeline, tissue sections are first stained with a dual-plex immunofluorescence assay to detect CD3 and LAG3, then imaged. The same sections are then stained with H&E and imaged a second time. Using an AI/ML approach applied to the H&E images, a first algorithm segments colon subregions (colonic crypts and lamina propria). A second algorithm then identifies CD3^+^/LAG3^+^ T-cells within the tissue compartments following image co-registration. Consequently, the method described takes advantage of both IF and brightfield imaging, where IF is used for cell/target identification and H&E is used for tissue segmentation and annotation. This technique, coupling two imaging techniques for the spatial identification of cells expressing a therapeutic target of interest, may shed light on responders versus non-responders in a clinical trial and may be useful in pre-screening of patients prior to treatment.

## Results

### Development of a dual-plex LAG3/CD3 chromogenic assay for digital image analytical quantification of CD3 and LAG3

Because of its prevalence as the ‘gold standard’ in clinical pathology, we first tested the suitability of a dual-plex CD3/LAG3 chromogenic IHC assay for downstream digital analysis. While dual chromogenic staining can enable simultaneous visualization of multiple markers, limitations exist in digital image analysis. For example, stain identification and isolation can be difficult in regions where signal colocalization and/or diffuse staining occurs. In contrast, chromogenic staining using brightfield illumination allows robust visualization of tissue structure and texture, two features that are advantageous when training AI/ML approaches for tissue segmentation. As such, we set out to determine if sufficient stain isolation for the two proteins of interest, could be conducted using a dual chromogenic staining approach, which would then allow the same brightfield illuminated images to be used for robust tissue segmentation using AI/ML. An initial approach combined yellow and purple chromogens to detect CD3 and LAG3, respectively. With this chromogen combination, target colocalization is predicted to result in a red signal, however the purple chromogen can often overshadow the yellow chromogen (Fig. S1). Unfortunately, the alkaline phosphatase (AP) signal amplification used in the yellow chromogen protocol resulted in a diffuse staining pattern for CD3, thus limiting downstream detection and co-localization analyses with the LAG3 stain. This diffuse staining pattern for CD3 was consistent across several AP-based chromogens, and of note, this staining increased the risk of false positive cell identification (Fig. S1). In response to this limitation, we substituted the AP-signal amplification step for a horseradish peroxidase (HRP)-based signal amplification (and subsequent HRP-based chromogen) in the CD3 staining protocol. This resulted in the specific staining for CD3 confined to T-cell plasma membranes, and LAG3 expression within the T-cell population, as expected. However, despite this optimization of the dual-plex staining protocol, downstream image analysis for CD3 and LAG3 proved problematic. Specifically, the still diffuse staining of CD3 critically increased the probability of false positive cell detection as the chromogen signal was more likely to be detected in the vicinity of CD3-negative cells, especially in hypercellular regions (Fig. S2). Pathologist review of this technique determined that it was inaccurate due to false positive identification, and therefore inadequate as a rapid, automated, whole slide image analysis method for the detection of CD3 and LAG3.

### Development of a robust dual-plex LAG3/CD3 immunofluorescent assay

**Figure 1:**
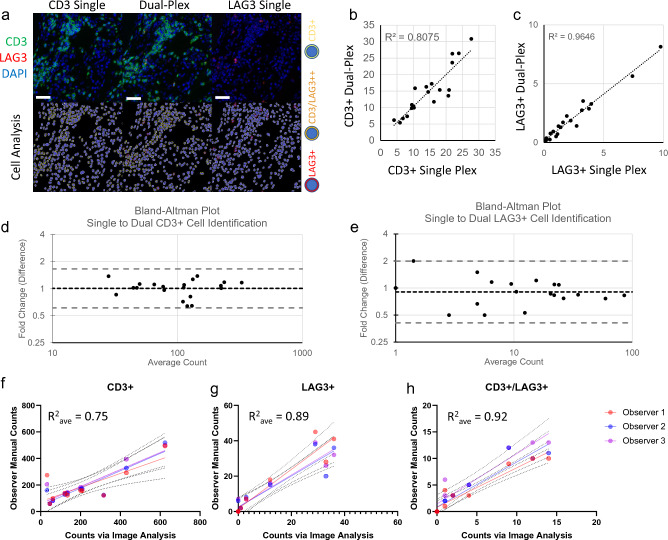
(**a**) Single plex assays for CD3 and LAG3 were compared to the dual plex assay to determine multiplex concordance with UC tissue shown in example (Top row, IF images; Bottom row, image analysis markup; Scale bars denote 50 μm). Image analysis for CD3+ cells (**b**,**d**) and LAG3+ cells (**c**,**e**) revealed strong concordance between assays. Image analysis was validated by comparing the algorithm to three trained and independent observers. This analysis revealed robust and repeatable image analysis algorithms for CD3+ (**f**), LAG3+ (**g**), and CD3+/LAG3+ dual positive (**h**) cells (**a**–**e**: N = 10 samples: 5 UC, 4 normal colon, 1 tonsil; **f**–**h** N = 10 samples all UC). Dashed lines denote 95% confidence intervals, colored by individual observer.

Due to the deficits of chromogen-based detection described above, we implemented a dual-plex immunofluorescence-based protocol for co-identification of CD3 and LAG3. To determine the robustness of the dual-plex assay, we tested positive and negative control tissues (Fig. S3), and the concordance of dual-plex IF assay to the individually validated single-plex IF-assays for each protein target. Three serial sections were mounted and stained for CD3, CD3/LAG3, and LAG3 respectively. Image analysis was conducted on co-registered regions of interest (ROIs) across the three tissue sections to determine concordance (Fig. 1a–e). In addition to development of a robust automated staining assay, a reliable downstream automated image analysis algorithm was necessary for semi-automation of the entire experimental pipeline. Using HALO image analysis software, cell identification based on the 2 fluorophores was conducted. DAPI was utilized for nuclear detection and implementation of a cell simulation approach. In cell simulation, the nuclear segmentation masks were dilated by a specified thickness (1 μm) and the region of dilation was analyzed as a surrogate for cell membrane or cytoplasmic staining. While this technique is difficult to apply to cell types with irregular morphology (e.g., fibroblasts), the identification of T-cells (with a largely rounded morphology) is well-suited for the implementation of this approach (Fig. 1a). To determine the accuracy of the algorithm, 10 human colorectal pinch biopsies were stained, imaged, and assessed by 3 trained, independent observers to first determine the ‘ground truth’. The three observers manually counted CD3^+^, LAG3^+^, and CD3^+^/LAG3^+^ cells within a randomly sampled grid covering at least 30% of the tissue area. These manual counts were then compared to the results from the automated image analysis algorithm. That algorithm was then manually tuned to minimize errors between the observations and automated cell counts (Fig. 1f–h, Fig. S4). Following tuning and implementation of the algorithm, the accuracy was assessed by a pathologist and deemed fit for purpose to identify CD3+ and LAG3+ cells in colorectal tissue.

### Increased localization of LAG3+ T-cells in Ulcerative Colitis

**Figure 2:**
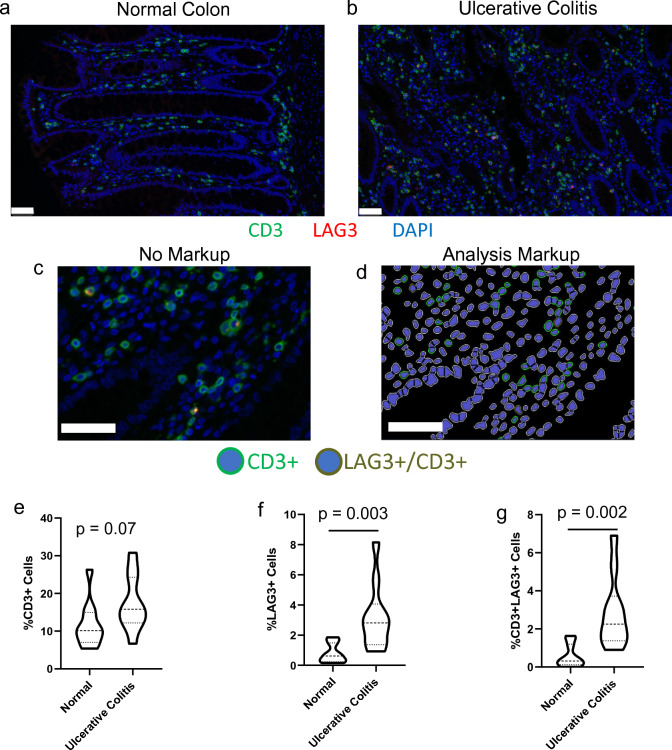
CD3+ and LAG3+ cells were identified in (**a**) normal and (**b**) ulcerative colitis colons using dual immunofluorescence and quantified using image analysis. (**c**) Example region of a UC sample with (**d**) corresponding analysis markup showing negative, CD3+, and CD3+/LAG3+ cells. While the percentage of cells positive for (**e**) CD3+ cells trended higher (p = 0.07), the proportion of (**f**) LAG3+ cells, and (**g**) CD3+/LAG3+ cells significantly increased (p = 0.003 and p = 0.0002, respectively). Graphs represent n = 10 specimens (5 normal, 5 UC; 2 analysis regions per sample). Scale bars denote 50 μm.

Using the validated staining procedure and image analysis algorithm, human colorectal tissue was assessed to determine alterations in T-cell localization in normal versus ulcerative colitis tissue (Fig. 2). In this analysis, 5 biopsies for each of normal tissue and ulcerative colitis were assessed in 2 ROIs of ~ 100,000 μm^2^ (average 784 cells/ROI, max: 1345 cells, min: 511 cells). Generally, there was an increased presence of T-cells in ulcerative colitis tissue compared to normal colon (Fig. 2a-b); however, when quantified via image analysis (Fig. 2c,d), this difference between groups was not statistically significant (Fig. 2e; 11.6% of cells normal colon, 17.4% of cells UC; p = 0.07). Interestingly, there was a substantial increase in LAG3+ cells (Fig. 2f; 0.80% of cells normal colon, 3.2% of cells UC; p = 0.0038), and CD3^+^/LAG3^+^ T-cells (Fig. 2g, 0.58% of cells in normal colon, 2.9% of cells UC, p = 0.0022). This finding indicated that the algorithm was sufficiently able to identify recently activated T-cells (i.e., LAG3^+^) that can contribute to the inflammatory cascade of UC. Further, visual inspection qualitatively revealed the potential to identify differential localization of LAG3^+^ cells in the colon sub-compartments. Generally, T-cells reside in the lamina propria with rare presence in the colonic crypts. However, in UC T-cells can be found within the crypts^13^. This finding is of importance due to their proximity to the gut space, and consequently, we sought to develop a technique to quantify the localization of CD3^+^ and LAG3^+^ T-cells within the colon sub-compartments.

### Development of an AI/ML algorithm for Ulcerative Colitis Tissue Segmentation

**Figure 3:**
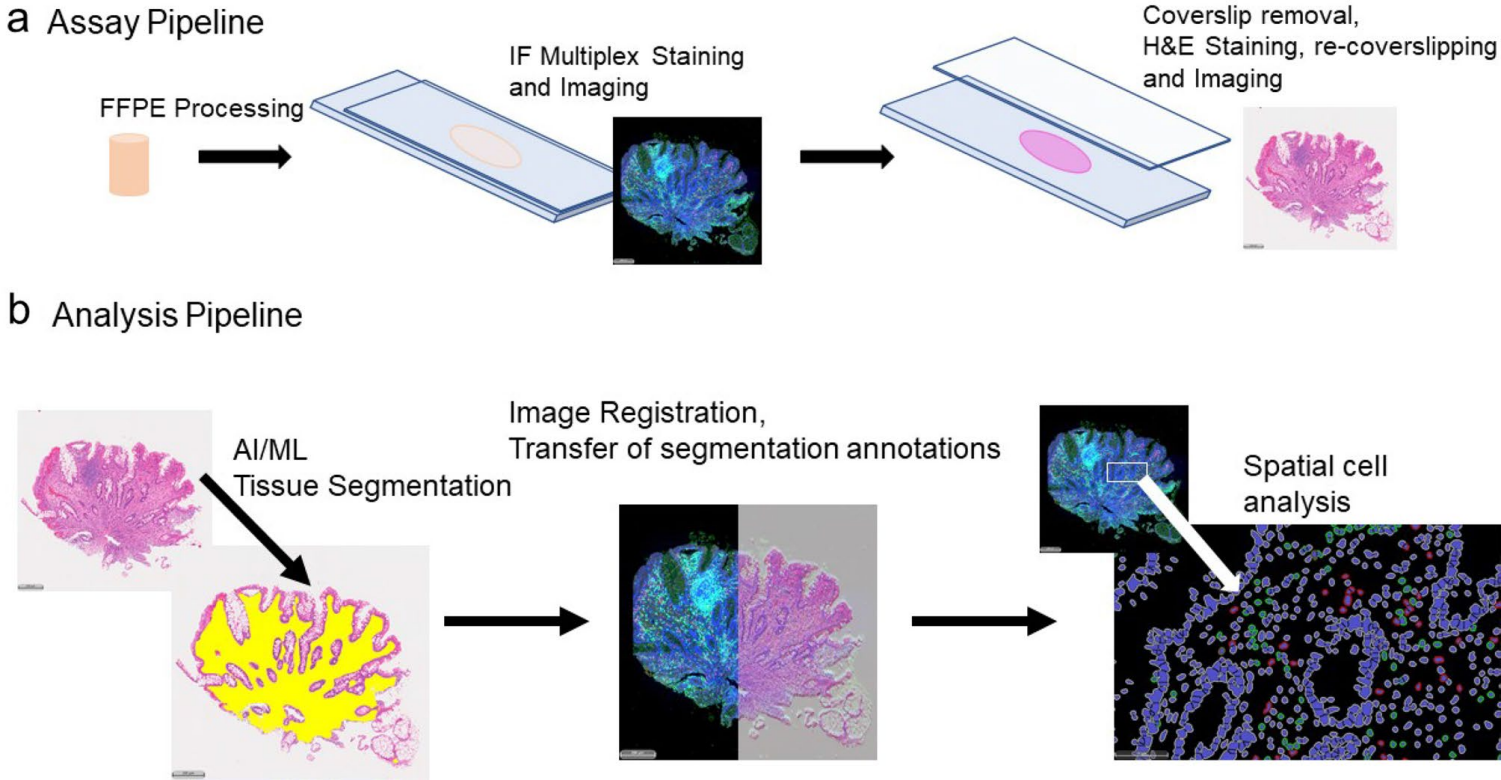
The semi-automated analysis process consisted of both an (**a**) assay pipeline and (**b**) analysis pipeline. (**a**) In the assay pipeline, FFPE tissue sections were stained using multiplex IF to detect CD3 and LAG3 with DAPI counter stain for nuclear detection. Subsequently, the coverslip was removed and the same sections were stained with H&E. Whole slide images of the IF and H&E stains were captured on a slide scanner capable of both fluorescence and brightfield imaging. (**b**) In the analysis pipeline, an AI/ML algorithm was implemented to segment the tissue sub-compartments based on the H&E image. The fluorescence image and brightfield images were co-registered using commercial software (Halo, Indica Labs). The same software was utilized to perform spatial cell analysis in annotated regions transferred from the AI/ML algorithm.

Preliminary efforts using DAPI-based tissue segmentation on the immunofluorescence CD3/LAG3 images were not adequate to accurately delineate the lamina propria and crypt tissue sub-compartments. As such, we tested a method where an AI/ML tissue segmentation algorithm was trained on H&E-stained UC tissue sections that had first been stained using the immunofluorescence protocol to detect CD3/LAG3 (Fig. 3). The assay was designed as follows; first, the tissue sections were stained using the multiplex IF protocol, and a whole slide image was captured. The same slides were then stained with H&E and imaged a second time. Importantly, the same slide scanner and objectives were used to capture both the IF and brightfield images. This technique enabled robust and repeatable image registration between the two slide scans (Fig. 3).

**Figure 4:**
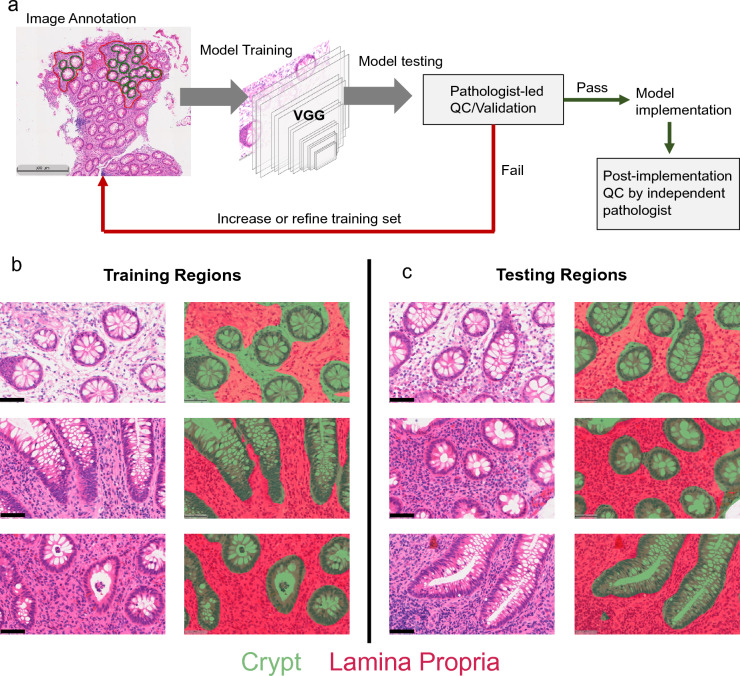
Development of an AI/ML algorithm for UC tissue segmentation. (**a**) The commercial image analysis software, HALO, was utilized for model development and implementation. In this process, H&E-stained images were annotated by a trained histology technician and these annotated images were fed into a built-in VGG-based algorithm. Following initial training, the accuracy of the model was assessed by a pathologist and either sent for additional annotation/refined training or passed along to model implementation. Following implementation, the model was validated by a second pathologist independent of the model training and initial testing. (**b**) Regions of tissue that were included in the annotations used in model training. (**c**) Regions of tissue segmented by the algorithm that were not included in the training set of annotations. In (**b**) and (**c**) the left column is the raw H&E image and the right column is the masked image following model implementation (green—crypt, red—lamina propria). Scale bars represent 500 μm (**a**) and 50 μm (**b**,**c**).

To develop an AI/ML tissue segmentation algorithm to delineate colonic crypts from the lamina propria, a VGG-16 (Visual Geometry Group) convolutional neural network (e.g.,^14^) was trained using Halo AI software. Briefly, nine H&E-stained UC specimens had portions of the tissue manually annotated (Fig. 4a) and these annotated regions were used for training the AI/ML algorithm. Since the algorithm is developed by minimizing cross entropy between the model and the training regions (Fig. 4b), it is important to validate accuracy on tissue not included in the ground truth of the algorithm (Fig. 4c). For algorithm validation, a pathologist assessment was utilized to determine the accuracy of the model. In an iterative approach, the model was updated or refined until the accuracy of the model passed the pathologist quality control assessment. While the present study focuses on the method and model development for deployment in a clinical trial (i.e., NCT03893565), it is important to note that the implementation of the model was independently validated by a second pathologist on the tissues in the clinical trial similarly to an independent test set. Additionally, to screen relevance across disease states, we tested the algorithm for both tissue segmentation and phenotyping on morphologically normal tissue to confirm the ground truth incorporated into the training and development of the phenotyping algorithm were able to accurately capture this tissue state (Figs. S5 and S6).

**Figure 5:**
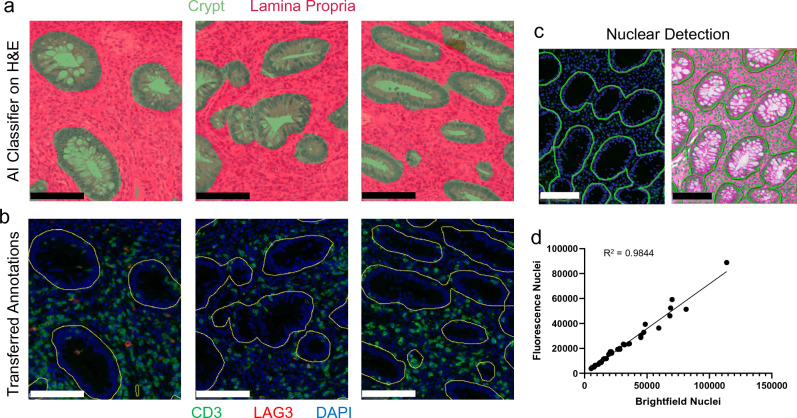
Image co-registration of the H&E image and corresponding CD3/LAG3 IF-stained images were conducted as described in Fig. 3 on UC samples. (**a**) AI/ML tissue classification was conducted on H&E-stained sections. (**b**) Following image registration, the annotations (yellow outlines) from the classifier were transferred to the fluorescence image enabling spatial identification of CD3/LAG3 cells. (**c**) Nuclei were identified via hematoxylin and DAPI in brightfield and fluorescence, respectively. Example images show markup for individual nuclei with blue and green overlays for fluorescence and brightfield respectively with colonic crypt regions shown outlined. (**d**) The number of nuclei were compared within annotation regions and exhibited a strong positive correlation (n = 36 annotated regions, 18 each for crypt and lamina propria). Scale bars represent 100 μm.

**Figure 6:**
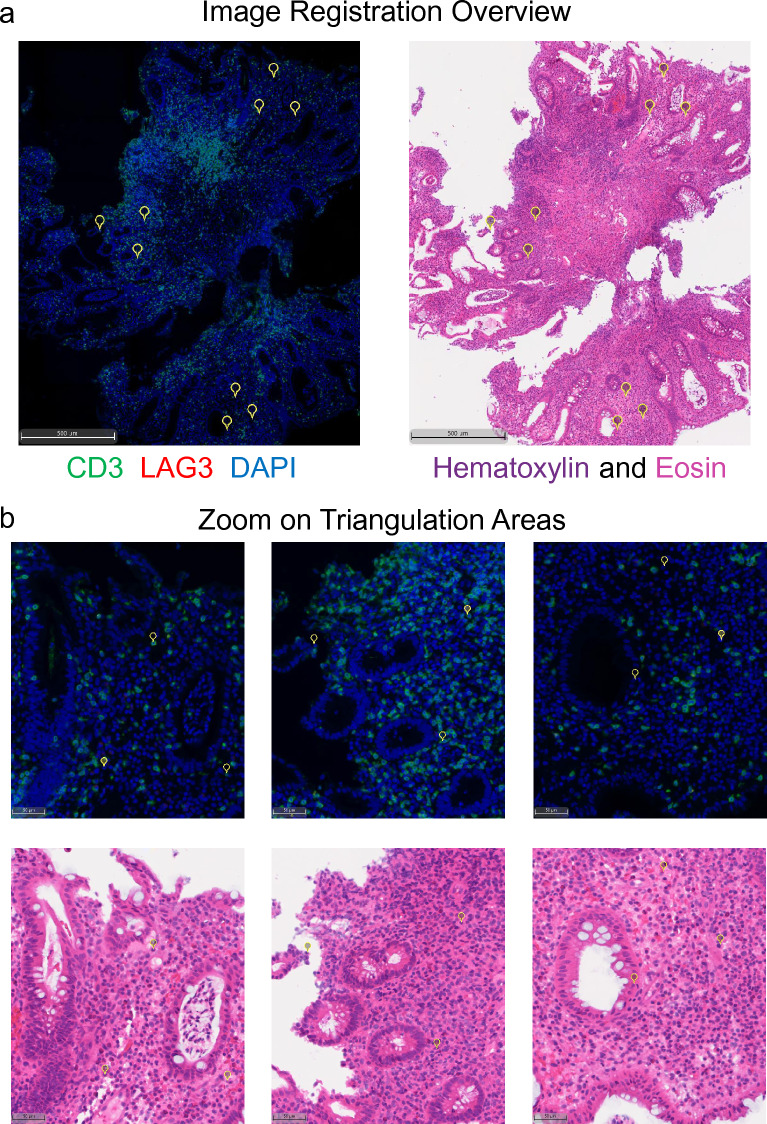
Assessment of image co-registration via triangulation points in UC tissue. (**a**) Triangulation landmarks were manually applied to the immunofluorescence image in Halo (3 sets of 3 points) and their corresponding locations were automatically applied to the H&E image. (**b**) Zoom in views on each of the three sets revealed co-registration was accurate to the single cell level.

Following the development of a robust and repeatable tissue segmentation algorithm that identified colonic crypts and lamina propria, the next step in the analysis pipeline was to transfer tissue masks generated from the H&E images (Fig. 5a) to the corresponding IF images for each tissue section (Fig. 5b) using image co-registration. Visual inspection of the image registration revealed accurate transfer of the masks to the IF images. Additionally, as a quantitative check, we performed nuclear segmentation based on the hematoxylin staining and the DAPI staining for the two images (Fig. 5c) and compared nuclear counts in the masked ROIs (Fig. 5d). Across all ROIs there was a strong positive correlation in the number of identified nuclei (R^2^ = 0.9844). As an additional check of image co-registration accuracy, triangulation landmarks were applied to an IF image in Halo image analysis software and corresponding landmarks were automatically applied to the corresponding H&E image. In this experiment, three sets of three landmarks each were used to triangulate at both high and low magnification. At low magnification (Fig. 6a), comparison of the triangulation areas revealed robust co-registration of the images across the tissue section. Additionally, inspection at high magnification (Fig. 6b) revealed co-registration was accurate down to the individual cell between the two images. This process was repeated in 6 more regions across two samples and the distances from the dropped pins to the expected sub-nuclear localizations were measured (Fig. S7). Across the 18 tests, the average distance between the dropped pin and the expected location (i.e., distance error of registration) was 2.49 ± 0.64 μm (mean ± standard deviation) indicating the error in image registration was less than the diameter of a single nucleus. It should be noted that this degree of accuracy in image co-registration would likely not be feasible with serial sections as nuclei move in and out of plane and section warping (i.e., swelling, wrinkling, or tearing) prior to mounting on a slide would limit accuracy between individual sections. Additionally, it is important to note that the accuracy of image co-registration (i.e., within 2.49 μm) is smaller than the thickness of a serial section.

### Implementation of Image Analysis Pipeline in Ulcerative Colitis Biopsies

**Figure 7:**
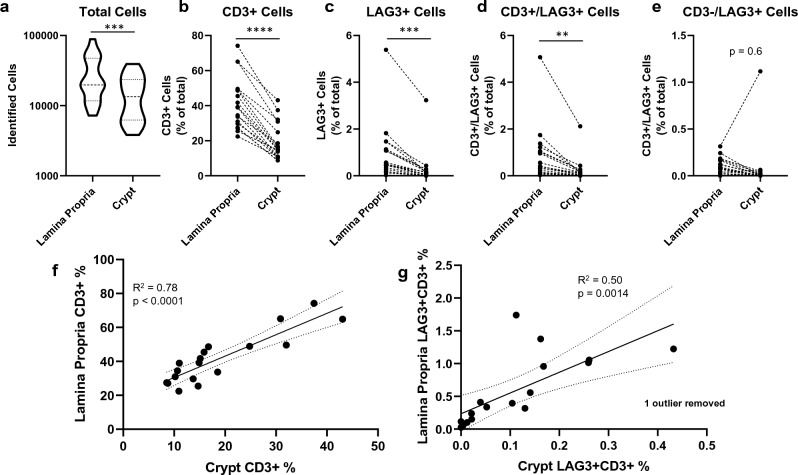
Implementation of the semi-automated pipeline on a cohort of ulcerative colitis biopsies. (**a**) total cells identified in the lamina propria and crypts. (**b**) CD3+, (**c**) LAG3+, (**d**) CD3+/LAG3+, (**e**) CD3-/LAG3+ cells identified within the tissue sub-compartments. (**f**) Correlation of CD3+ T-cells in the lamina propria versus crypt. (**g**) Correlation of CD3+/LAG3+ cells in the lamina propria versus crypt (one subject was removed due to high false positive LAG3 identification, Grubbs’ test: G = 3.893). N = 18 pinch biopsies, ** denotes p, 0.01, *** p < 0.001, **** p < 0.0001.

Following validation, the pipeline described above (Fig. 3) was implemented on ulcerative colitis pinch biopsies (n = 18) that were not included in the assay and image analysis validations. The semi-automated process enabled spatial quantification of total number of cells, CD3^+^ cells, LAG3^+^ cells, CD3^+^/LAG3^+^ cells, and CD3^-^/LAG3^+^ cells. Due to the size and cellularity of the biopsies, the variation in number of cells identified in both the lamina propria and the crypts spanned an order of magnitude across the cohort (Fig. 7a). Further, greater numbers of cells were identified in the lamina propria compared to the crypts largely due to comparatively higher tissue area covered by the lamina propria. Generally, the pipeline identified T-cells in the lamina propria with high subject variability between ~ 20% and ~ 80% of cells presenting CD3. In the crypts, T-cells were less abundant accounting for ~ 10% to ~ 40% of identified cells (Fig. 7b). As LAG3^+^ cells should represent a subset of CD3^+^ cells, LAG3^+^ cells were found to be less abundant than CD3^+^ cells, accounting for up to 5% cells in the lamina propria and 3% of cells in the crypts (Fig. 7c). In most cases CD3^+^/LAG3^+^ cells accounted for the majority of LAG3^+^ cells identified by the algorithms (Fig. 7d). However, one case identified a substantial proportion of cells identified as LAG3^+^ that were CD3^-^ (Fig. 7e). This biologically unexpected finding is addressed in the subsequent section. Comparing the tissue sub-regions, the number of identified CD3^+^ cells in the colonic crypts correlated with the number of CD3+ cells in the lamina propria (R^2^ = 0.78), indicating the presence of T-cells in the crypt may be associated with the total number of T-cells identified within these study samples (Fig. 7f). However, the localization of CD3^+^/LAG3^+^ cells was more spatially variable. In several instances, the presence of CD3^+^/LAG3^+^ cells in the lamina propria diverged from the presence of CD3^+^/LAG3^+^ cells in the colonic crypts (Fig. 7g). Specifically, there were instances where high numbers of CD3^+^/LAG3^+^ cells in the lamina propria did not correspond to high numbers of CD3^+^/LAG3^+^ cells in the crypt. Consequently, this method of spatial identification of LAG3^+^ T-cells provides additional information not readily available from raw cell counts. That is, the total number of LAG3^+^ T-cells within colonic crypts is not predictable on a single patient level based on total LAG3^+^ T-cells in a tissue section. As such, this spatial information may inform clinical efficacy or patient screening where cell localization is of interest to clinical trial development and analysis. In short, this assay may provide information that general cell population analyses cannot.

### Advantages of a CD3/LAG3 multiplex IF assay to limit false positive detection

An added advantage of the dual-stained (IF and H&E) approach used in this study is that an IF multiplex image can be directly compared to its corresponding H&E image for elucidating tissue morphology. This method can provide a higher degree of accuracy over separate IF and H&E stains using serial sections, especially when there is a need to interrogate the spatial composition of low abundance cell types. As mentioned above, we identified one case where a substantial proportion of LAG3^+^ cells were CD3^-^ (Fig. 7e). While other cell types besides CD3+ T-cells may express LAG3 (e.g., Natural Killer cells,^15^), visual inspection of the H&E staining for this sample identified abundant eosinophil infiltration. Eosinophils are known to be autofluorescent and are often identified as such in flow cytometry using gated autofluorescence forward scatter^16,17^. Additionally, eosinophils are known to be active in subsets of UC patients^18^. Image registration between the IF and H&E images revealed that much of the signal in the LAG3 channel could be attributed to eosinophils (Fig. S8). This finding indicates the importance of assaying CD3 and LAG3 co-expression to identify true LAG3^+^ T-cells. Because an abundance of eosinophils can overshadow rare cell populations like LAG3^+^ T-cells, multiplexing with CD3 and solely counting the double positive cells can mitigate false positives that could occur by counting solely LAG3^+^ cells. As such the combination of these two markers will enable the identification of T-cells expressing LAG3^+^, but other potential cell types expressing LAG3^+^ will not be identified.

### Outlook

Here, we describe the development of a novel assay for spatial identification of LAG3^+^ T-cells in UC biopsies. LAG3^+^ T-cells are associated with active inflammation in UC and pose a viable target for immunotherapies^2,4,6^. While previous work in flow cytometry and gene expression have identified LAG3 as a potential biomarker and therapeutic target^4^, those assays need additional steps in handling and sample processing that may fall outside the remit of standard practices while biopsy collection and paraffin processing remain standard. In addition to relying on standard sample handling techniques, the spatial information preserved by this technique may add valuable insight into both the treatment and disease progression of lesions. For example, oral administration of a LAG3 targeted therapy may localize better to the colonic crypts and be less reliant on passive drug transport mechanisms to reach the targeted cell population. While T-cell localization in the lamina propria was associated with T-cells in the colonic crypts, the presence of LAG3^+^ cells were less predictable without the spatial information afforded by the present method (Fig. 7f,g). This method enables robust identification of spatially-resolved LAG3^+^ T-cells in UC and may guide future patient selection for clinical trials targeting this disease. Looking forward, a limitation of this assay is its reliance on multi-step staining and imaging for assay automation. Future efforts may be targeted at incorporating additional morphology markers into the IF panel (e.g., a pan cytokeratin marker) to enable robust tissue segmentation using the IF image in the absence of the H&E stain. Further, since the ground truth of the algorithm training did not contain potential variations in biology due to a LAG3-targeting therapy, independent validation of the model upon implementation is recommended. In this case, a second independent pathologist determined accuracy of the model upon implementation in a clinical trial. Additionally, future efforts may seek to incorporate the spatial presence of CD3^+^/LAG3^+^ cells in the context of disease state, demographic data, and treatment response to better understand disease progression and treatment potential.

## Materials and methods

### Method overview

Human biological samples were sourced ethically from commercial vendors (BioIVT/Asterand and Discovery Life Sciences) that have been approved by GSK human biological sample management ethical committee. Prior to sample acquisition informed consent for general research was obtained by commercial vendors that covered the research conducted in this study. All methods and validation processes followed GSK Standard Operating Procedures with standardization of instrument calibration and qualification where records of repairs and routine maintenance, and any non-routine work, are tracked. Automated instrumentation, quality-controlled reagents, and control slides were used to identify run-to-run and intra-run variability. FFPE tissue blocks, normal colon (n = 10), ulcerative colitis (n = 25) and control tissues (tonsil, n = 2; skeletal muscle, n = 7) were obtained from various commercial sources.

All immunohistochemical chromogenic (IHC) and immunofluorescence (IF) assays were performed on the Ventana Discovery Ultra automated system (Ventana, Tucson, AZ), using Ventana reagents. Whole slide images were captured using the Vectra Polaris slide scanner (Akoya Biosciences, Marlborough, MA). H&E staining was performed using standard protocols on the Symphony automated staining platform (Ventana, Tucson, AZ). Digital image analyses were performed using HALO image analysis software (Indica Labs, Albuquerque, NM).

### Dual-plex immunohistochemistry

IHC assays were carried out on the automated Ventana Discovery Ultra autostainer. Briefly, 3.5 µm sections were prepared from FFPE tissue blocks and mounted onto Autofrost IHC glass slides and dried overnight at ambient conditions. All slide processing with the exception of coverslipping was performed on the Discovery Ultra. Deparaffinization followed by heat induced epitope retrieval was performed using Tris-based (EDTA) buffer solution (CC1, Ventana, Tucson, AZ). Prior to initial primary antibody incubation, Discovery Inhibitor and Discovery Antibody Block were applied to each slide for peroxidase quenching and blocking non-specific binding, respectively. The mouse anti-human LAG3, Clone 17B4 (LifeSpan BioSciences Inc., Seattle WA) was added and incubated for 32 min at 36 °C at 1 µg/mL. Detection of the mouse primary antibodies were performed by a 12-min incubation at 36 °C followed by the anti-Mouse HQ conjugated mAb and a 12-min incubation with anti HQ-HRP (both from Ventana, Tucson, AZ). Chromogenic detection was achieved by application of Discovery Purple (Ventana, Tucson, AZ) for 16 min. Other chromogens were assessed but not reported in the present manuscript. Slides were rinsed and the pre-diluted rabbit anti-human CD3, Clone 2GV6 (Ventana, Tucson, AZ) was applied and incubated for 32 min at 36 °C. Detection of the rabbit primary antibodies were performed by a 16-min incubation at 36 °C with anti-Rabbit NP followed by a 16-min incubation with anti NP-AP (both from Ventana, Tucson, AZ). Chromogenic detection was achieved by application of Discovery Yellow (Ventana, Tucson, AZ) for 16 min. Tissue sections were counterstained with hematoxylin II and bluing reagent (both from Ventana, Tuscon, AZ) for 4 min each. Following washing, dehydration, and clearing, slides were coverslipped using the Ventana Symphony (Ventana, Tucson, AZ).

### Dual-plex immunofluorescence

Immunofluorescence was performed on 3.5 µm sections prepared similarly to the description above. Briefly, deparaffinization followed by heat induced epitope retrieval (HIER) was conducted using Tris-based (EDTA) buffer solution, CC1 at 97 °C for 8 min. Discovery Inhibitor (Ventana, Tuscon, AZ) was applied and incubated for 12 min, followed by a 12 min incubation with Antibody Block (Ventana, Tuscon, AZ). The primary anti-LAG3 mouse mAb (17B4 LifeSpan BioSciences) was added and incubated for 32 min at 36 °C at 1 µg/mL, followed by a 16 min incubation with the anti-mouse-HQ conjugated antibody (Ventana, Tucson, AZ) at 36 °C. Anti-HQ-HRP (Ventana, Tucson, AZ) was applied and incubated for 16 min at 36 °C. Immunofluorescent detection was achieved by the application of Discovery Cy5 (Ventana, Tucson, AZ) for 4 min followed by Cy5 H2O2 (Ventana, Tucson, AZ) for 1 h and 32 min. A second HIER step was applied for 16 min at 91 °C using a citrate-based solution (CC2, Ventana, Tucson, AZ). DISC Inhibitor was added and incubated for 8 min, followed by the second primary antibody, anti-CD3 rabbit mAb (2GV6, Ventana) at 0.4 µg/mL for 32 min at 36 °C. Anti-Rabbit-HQ conjugated antibody (Ventana, Tucson, AZ) was applied for 16 min at 36 °C, followed by anti-HQ-HRP (Ventana, Tucson, AZ) application for 16 min at 36 °C. Immunofluorescent detection was achieved by Rhodamine 6G (Ventana, Tucson, AZ) for 4 min followed by Rho 6G H2O2 (Ventana, Tucson, AZ) for 1 h and 32 min. Tissue sections were counterstained with Spectral DAPI (Akoya Biosciences, Marlborough, MA) for 32 min, washed, and coverslipped using Prolong Gold (Invitrogen, Carlsbad, CA). Slides were scanned using optimized exposure settings on the Akoya Vectra Polaris (Malborough, MA) whole slide scanner.

### H&E tissue staining

Following whole slide scan of the IF-stained samples, coverslips were carefully removed. Slides were then stained using a standard H&E protocol on the Symphony automated staining platform (Ventana). Slides were then coverslipped a second time and whole slide scans were conducted using the same slide scanner (Polaris) used in the above assay.

### Image acquisition and analysis

HALO image analysis software (v2.3 and v3.5) was used for all analyses. The H&E tissue classifier was trained by manual annotation of crypt and lamina propria regions of the tissues and trained using a VGG-based algorithm. The training resulted in a semantic segmentation algorithm that was qualified as fit-for-purpose via an iterative approach overseen by a pathologist. As the model was passed through training steps, the pathologist would review the model accuracy and either annotations used in the training would be altered or further training would be applied. Upon model implementation on clinical trial samples, a second independent pathologist oversaw accuracy in an independent testing of the model.

Upon H&E image segmentation based on the machine learning algorithm, the tissue classification was transferred to the immunofluorescence whole slide scans. Co-registration of IF and H&E images was conducted via co-registration procedures built into the analysis software and was visually inspected for accuracy. As a second check of registration accuracy, the number of nuclei based on both DAPI and hematoxylin staining were counted via nuclear segmentation algorithms applied to the IF and H&E images, respectively.

Identification of individual cells and cells positive for markers of interest was conducted using a cell simulation-based phenotyping algorithm. Briefly, individual cells were identified via segmentation based on DAPI signal and declumping/object separation using Halo algorithms with small and large object discard thresholds at 11 and 550 μm, respectively. Following identification of individual nuclei, cell body/membranes were simulated by dilating the nuclear objects by 1 μm and subtracting the nuclear objects leaving an annular object for each cell representing the cell body/membrane. The average fluorescent signals were then calculated across the pixels representing the cell body and cutoff thresholds were identified (e.g., 36 and 19 for CD3 and LAG3, respectively on the 0–255 scale). Accuracy of the algorithm was calculated by comparing algorithm counts to the positive cells identified by 3 independent observers.

### Statistical analysis

Statistical analyses were conducted, and graphs were developed using Graphpad Prism 8. Linear regressions are depicted along with R^2^. In certain cases, 95% confidence intervals are included (dashed lines) and p values denoting non-zero slope of the linear regression. For nuclear count data, a linear Poisson regression was conducted and pseudoR^2^ is presented. Analyses comparing normal and UC tissue were conducted using a non-paired two-tailed t-test. Analyses comparing crypt and lamina propria in UC samples were assessed using a paired (crypt vs lamina propria within individual specimens) two-tailed t-test. Where appropriate, outliers were identified with a Grubbs’ test. A significance threshold (denoted by *) was set at p < 0.05. Notations for other p value thresholds are noted in figure captions.

### Supplementary Information

Supplementary Figures.

## Data Availability

The authors confirm that the data supporting the findings of this study are available within the article and its supplementary materials. Access to additional raw data (e.g., images) and Halo algorithms are available upon reasonable request to the corresponding author.
