# Supplementary material for: A machine learning approach toward automating spatial identification of LAG3+/CD3+ cells in ulcerative colitis
Source: Sci Rep. 2023 Dec 8;13:21759. doi: 10.1038/s41598-023-49163-5 (PMC10709428; doi:10.1038/s41598-023-49163-5)
Supplement: Supplementary file 1 — Supplementary Figures. [file 41598_2023_49163_MOESM1_ESM.docx]

**SUPPLEMENTAL INFORMATION**

**A Machine Learning Approach toward Automating Spatial Identification of LAG3+/CD3+ Cells in Ulcerative Colitis**

Edward D. Bonnevie^1*#^, Eric Dobrzynski^1*^, Dylan Steiner^1^, Deon Hildebrand^2^, James Monslow^1^, Mohan Singh^1^, Vilma Decman^1^, David Krull^1^

**
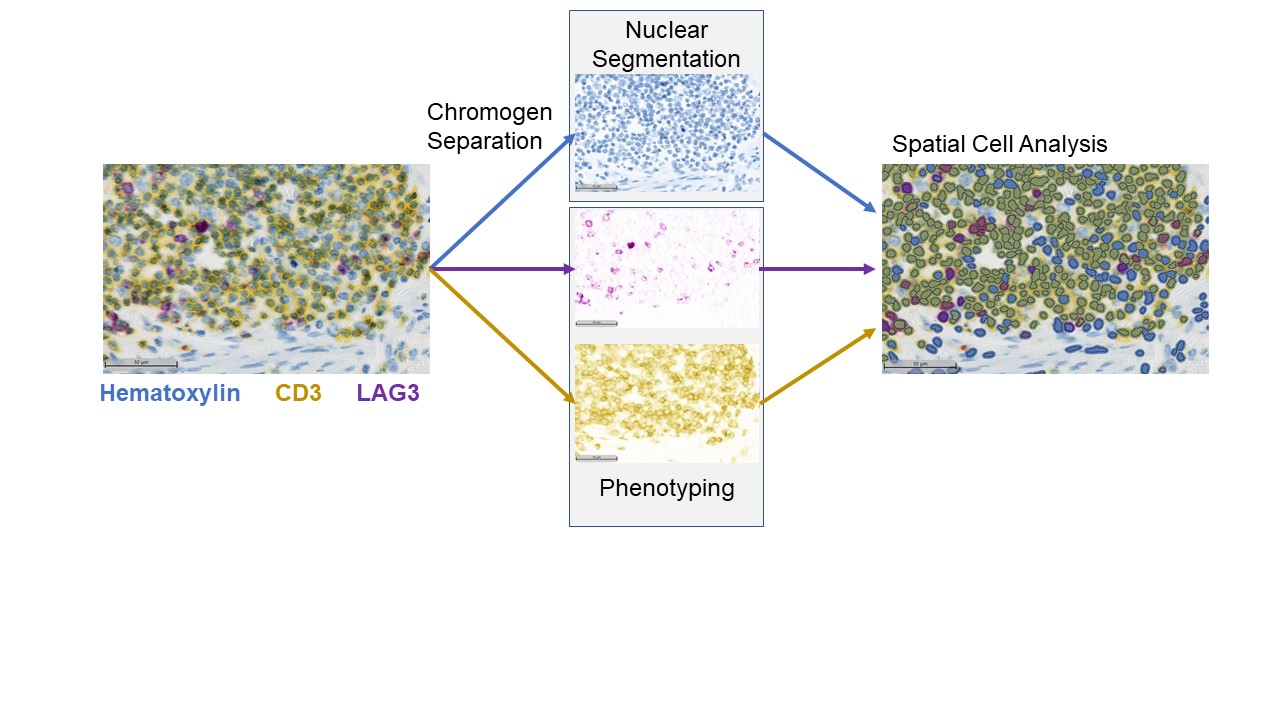
**

**Supplemental Figure 1. Chromogen staining and image analysis.**

**
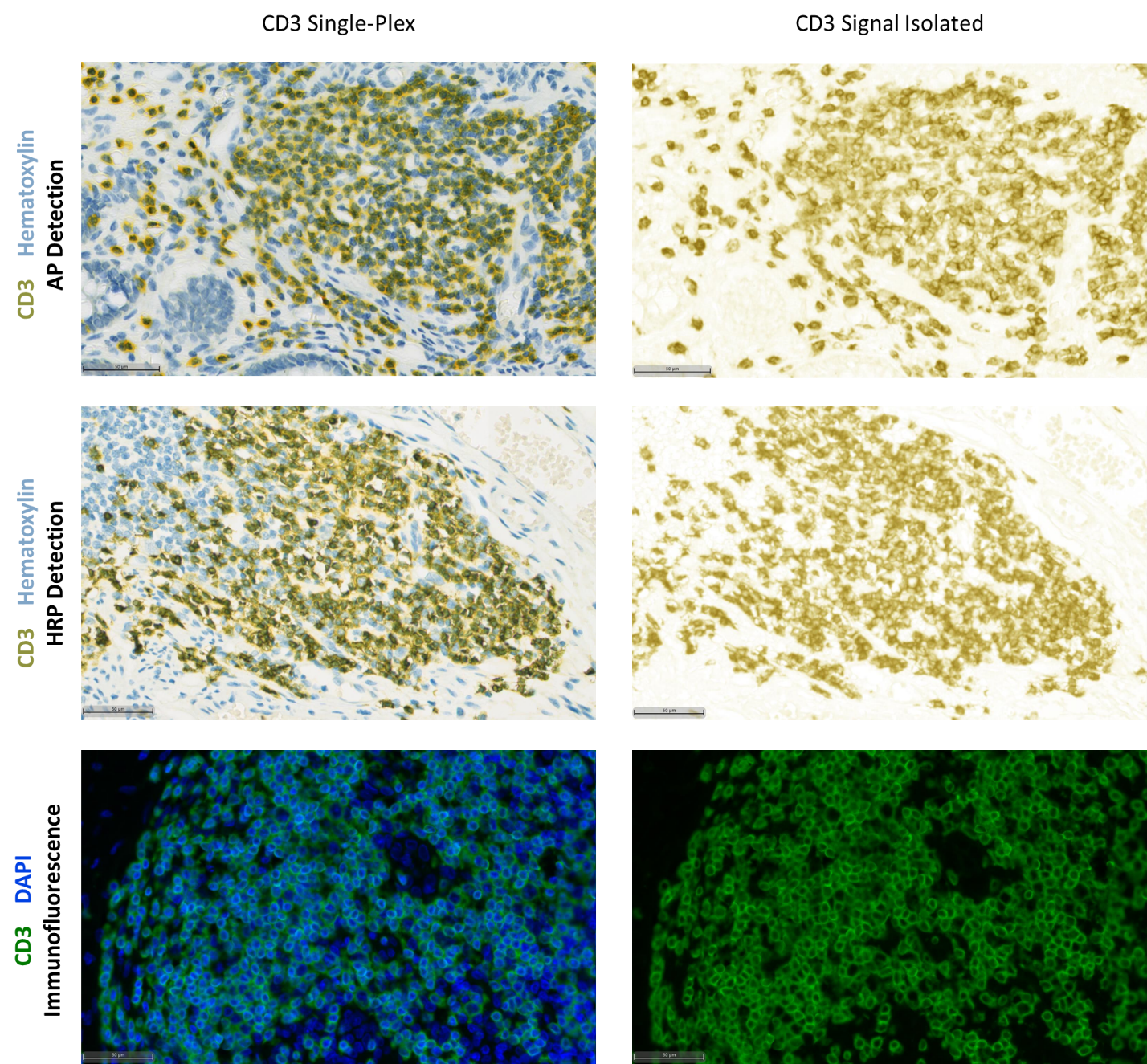
**

**Supplemental Figure 2: Comparison of CD3 signal in IHC assays (AP and HRP detection) to IF assay in UC tissue. Diffuse pericellular staining in IHC assays limited algorithm accuracy.**

**
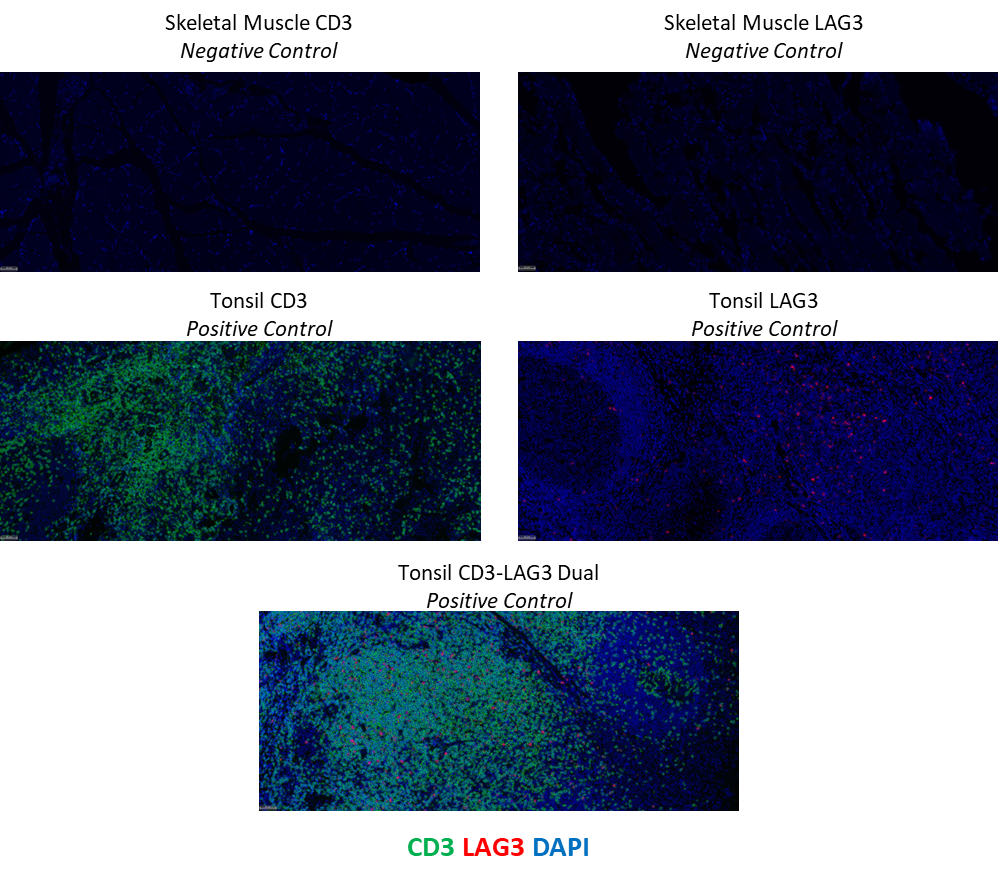
**

**Supplemental Figure 3: Positive and Negative control tissue staining for single-plex, and positive control tissue for dual plex assay.**


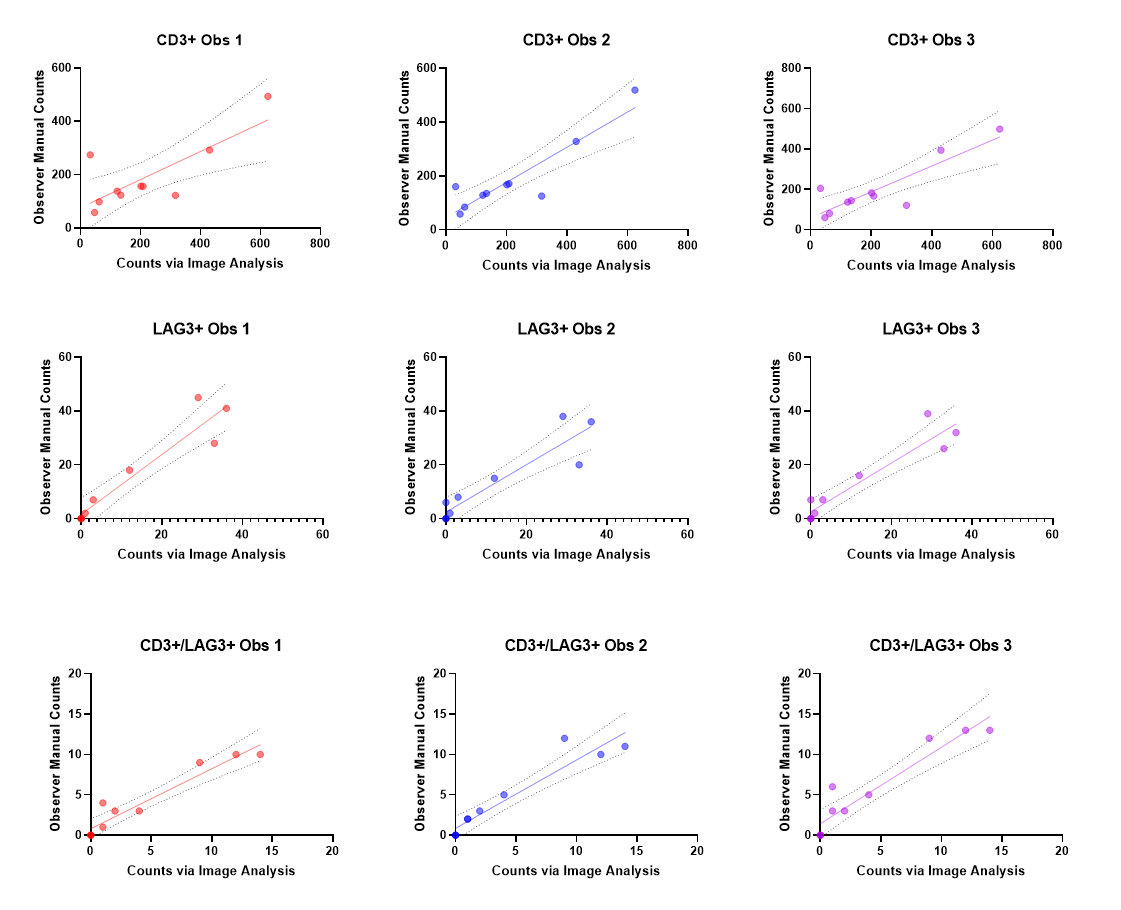


**Supplemental Figure 4: Results comparing image analysis and manual observer counts separated by individual observer (obs) with 95% confidence intervals shown as dashed lines.**


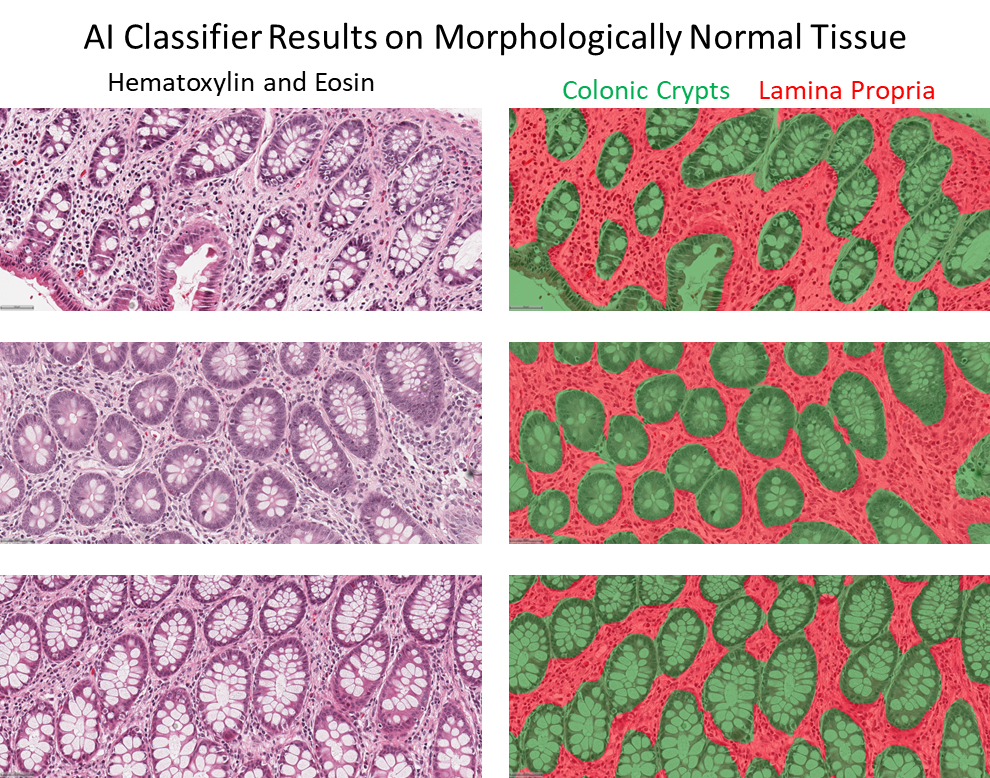


**Supplemental Figure 5: Results of AI-based tissue classifier on morphologically normal tissue.**

**
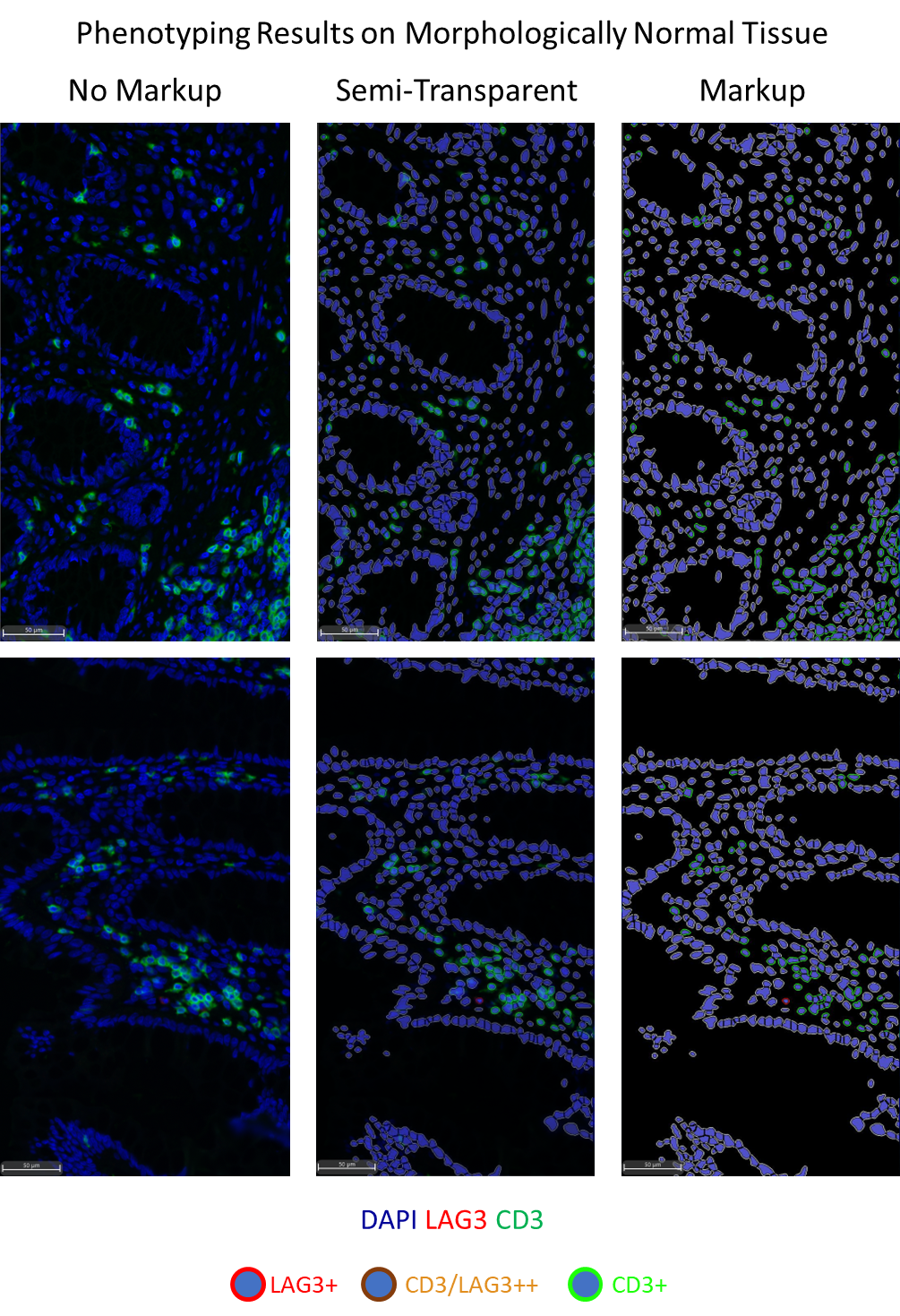
**

**Supplemental Figure 6: Results of phenotyping algorithm on morphologically normal tissue.**

**
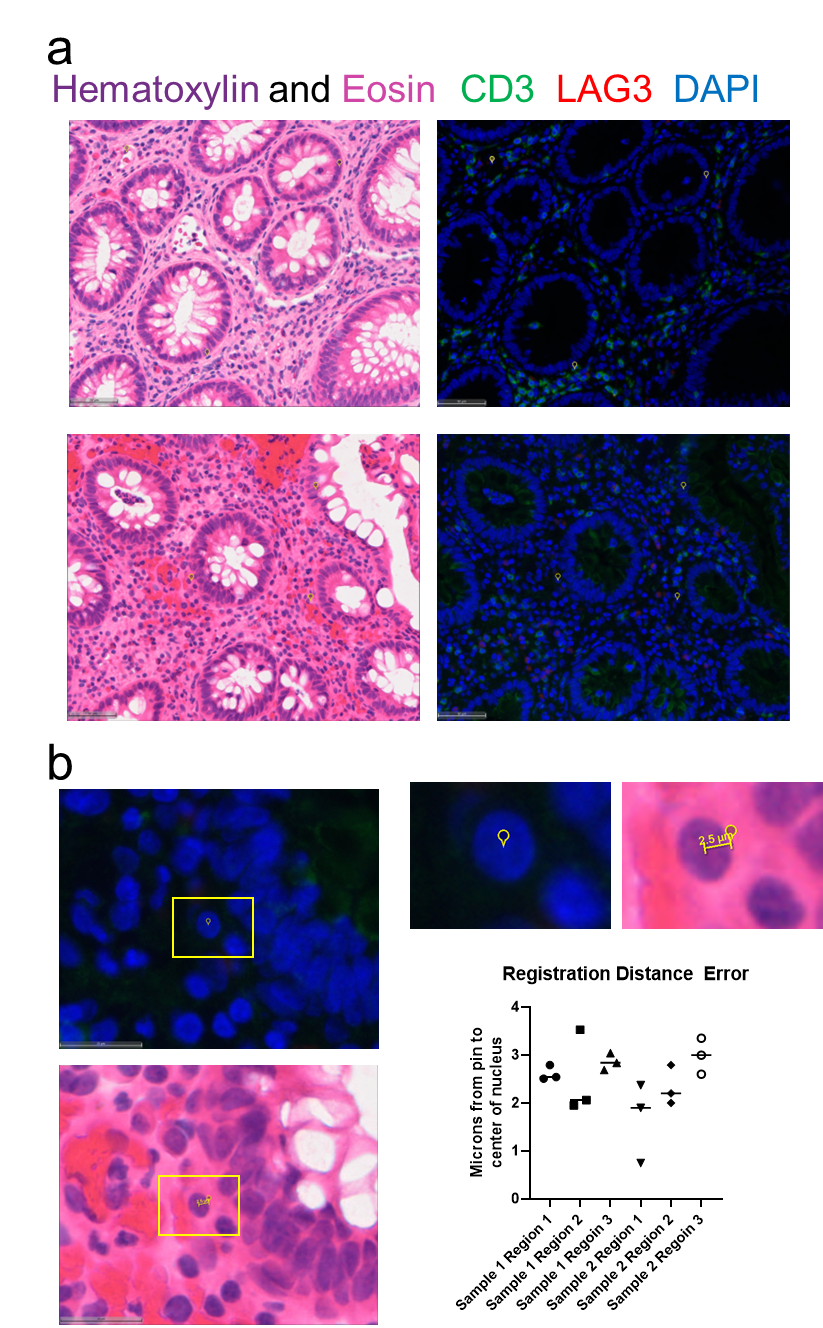
**

**Supplemental Figure 7: Results of quantifying triangulation errors. (a) Example H&E and IF images with regions where three pins were dropped on the IF image and the pins were automatically applied to the H&E images via image registration. (b) Quantification of the error (i.e., distance the pin was mis-applied to the H&E image) in co-registration across three regions with three pins for two samples. Yellow boxes denote zoom-in regions.**

**
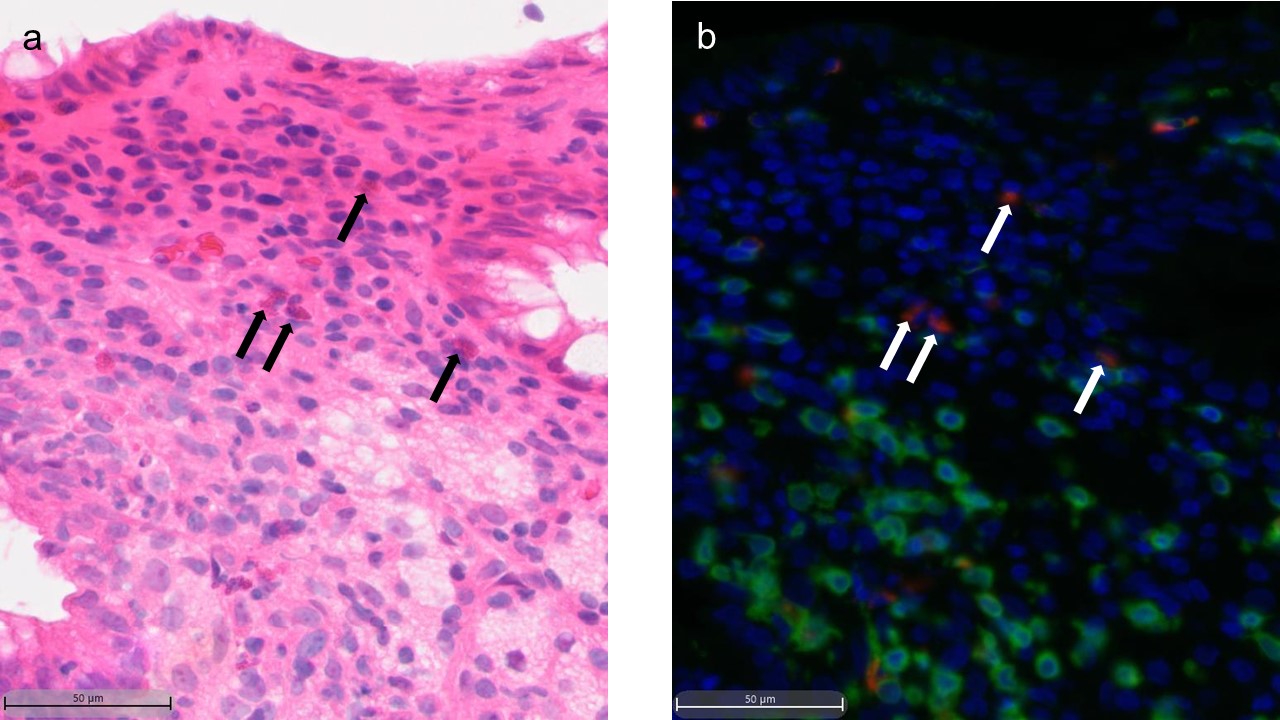
**

**Supplemental Figure 8: Eosinophils (black arrows) identified in the H&E image (a) showing a false-positive signal for LAG3 (white arrows) in the corresponding multiplex IF image for LAG3/CD3.**
